# The relationship between maternal age, obesity and child mortality: a cross-sectional study using 2013–2014 Demographic and Health Survey in Democratic Republic of the Congo at national, and sub-national levels

**DOI:** 10.1017/S1368980024002647

**Published:** 2025-01-07

**Authors:** Zacharie Tsala Dimbuene, Raphaël Muanza Nzuzi, Severin Mabanza Matondo

**Affiliations:** 1 School of Population and Development Studies, University of Kinshasa, Kinshasa, Democratic Republic of the Congo; 2 Faculty of Economics and Management, University of Kinshasa, Kinshasa, Democratic Republic of the Congo

**Keywords:** Maternal obesity, Child health, Mortality, Democratic Republic of the Congo

## Abstract

**Objective:**

To investigate the relationship between maternal age and nutritional status, and test associations between maternal nutritional status and child mortality with a focus on maternal obesity.

**Design:**

Secondary analysis of data from nationally representative cross-sectional sample of women of reproductive ages (15–49 years) and their children under 5 years. The outcome variable for maternal nutritional status was BMI, classified into underweight (BMI < 18·50 kg/m^2^), normal weight (18·50–24·99 kg/m^2^), overweight (25·0–29·9 kg/m^2^) and obesity (>=30·0 kg/m^2^). Child mortality was captured with five binary variables measuring the risk of dying in specific age intervals (neonatal, post-neonatal, infant, childhood and under-five mortality).

**Setting:**

The most recent Demographic and Health Surveys from Democratic Republic of Congo (DRC).

**Participants:**

The final samples consisted of 7892 women of reproductive ages (15–49 years) and 19 003 children aged 0–59 months.

**Results:**

The prevalence of obesity was estimated at 3·4 %; it increased with maternal age. Furthermore, obesity unevenly affected provinces in the Democratic Republic of the Congo: Kinshasa, South Kivu, North Kivu and Maniema were most affected. Finally, maternal obesity showed mixed effects on child mortality.

**Conclusion:**

The prevalence of obesity is still low; however, provinces are unevenly affected. Therefore, interventions and programmes to improve nutrition should incorporate geographical disparities to tackle adverse child outcomes associated with maternal obesity, to limit negative consequences of maternal obesity, including non-communicable diseases which might be a strong impediment to reach Sustainable Development Goals (SDG) 2 and 3.

Most epidemiologists trace the **origins of obesity** back in the 1970s^(1)^; during that time, it was mostly confined to developed countries^(2,3)^. Very recently, obesity significantly spanned in developing countries^(4–6)^. In the 2000s, the WHO recognised obesity as a global epidemic^(7)^ because of its interlinkages with non-communicable diseases (NCD). Recent estimates indicated that 1·5 billion of adults aged 20 years and above were overweight; among them, over 200 million of men and 300 million of women were obese^(5)^. Previous studies showed strong variations of obesity across regions and countries, with sub-Saharan Africa (SSA) being the less affected region^(8)^.

Research has established a relationship between maternal age and maternal obesity^(9)^; however, this relationship is not well documented in developing countries. There are plausible reasons to suggest that maternal obesity may increase with age. First, studies consistently showed that women gain weight during pregnancies^(10–12)^ and weight gain persists after deliveries. Second, parity increases with maternal age, making weight gain more likely. According to 2022 Global Nutrition Report^(13)^, even though the prevalence of obesity is lower than the regional averages (20·8 % for women and 9·2 % for men), a sizeable percentage of people aged 18 years and over in Democratic Republic of the Congo (DRC) are living with obesity (11·6 % and 4·5 % of women and men, respectively). Furthermore, evidence indicated that the prevalence of overweight among adult aged 18 years and over increased from 18·8 % to 20·6 % between 2010 and 2014, while the prevalence of obesity rose from 3·7 % to 4·4 % during the same period. These figures, however, mask regional and local disparities of obesity^(14–16)^. For instance, a localised study among a mine-based workforce showed that the prevalence of obesity increased from 4·5 % to 11·1 %^(15)^. Therefore, the first objective of this paper is to estimate the probabilities of maternal nutritional status using BMI and adopting a sub-national perspective and emphasising urban–rural differences and poverty effects.

Although evidence showed that under-five mortality (U5M) rates in DRC has declined from 186 p. 1000 in early 1990s to 81 p. 1000 in 2020^(17,18)^, there still are provincial disparities in the country^(19)^. In spite of the decline of U5M, DRC accounts for 11 % of annual deaths of children under 5 years in SSA^(18)^. Previous research has identified several factors at different layers (child, maternal and community levels) associated with U5M^(20)^, including maternal obesity^(11,12,21–25)^. Consequences of maternal obesity of child health outcomes include preterm births^(26)^: fetal deaths, stillbirths and infant deaths^(12,21,25,27,28)^. Yet, this relationship is poorly documented in developing countries, including DRC. Since maternal obesity is alarmingly increasing in developing countries, and child health is still poor in these settings, it is crucial to scrutinise the relationship between maternal obesity and pregnancy and child health outcomes. Therefore, the second objective of the paper is to unpack the relationship between maternal obesity and child mortality at national and sub-national levels.

## Methods

### Data source

The paper utilises data from the 2013–2014 Demographic and Health Surveys (DHS) conducted in the Democratic Republic of Congo. DHS are nationally representative surveys, using a two-stage sampling design, which collected information on households, women and men of reproductive ages, anthropometric measures, contraception and family planning, among others. All men and women aged 15–59 and 15–49 years, respectively, in the selected households were eligible to participate in the survey if they were either usual residents of the household or visitors present in the household on the night before the survey. This paper reports on findings from women of reproductive ages in the individual record files.

**Figure 1.**
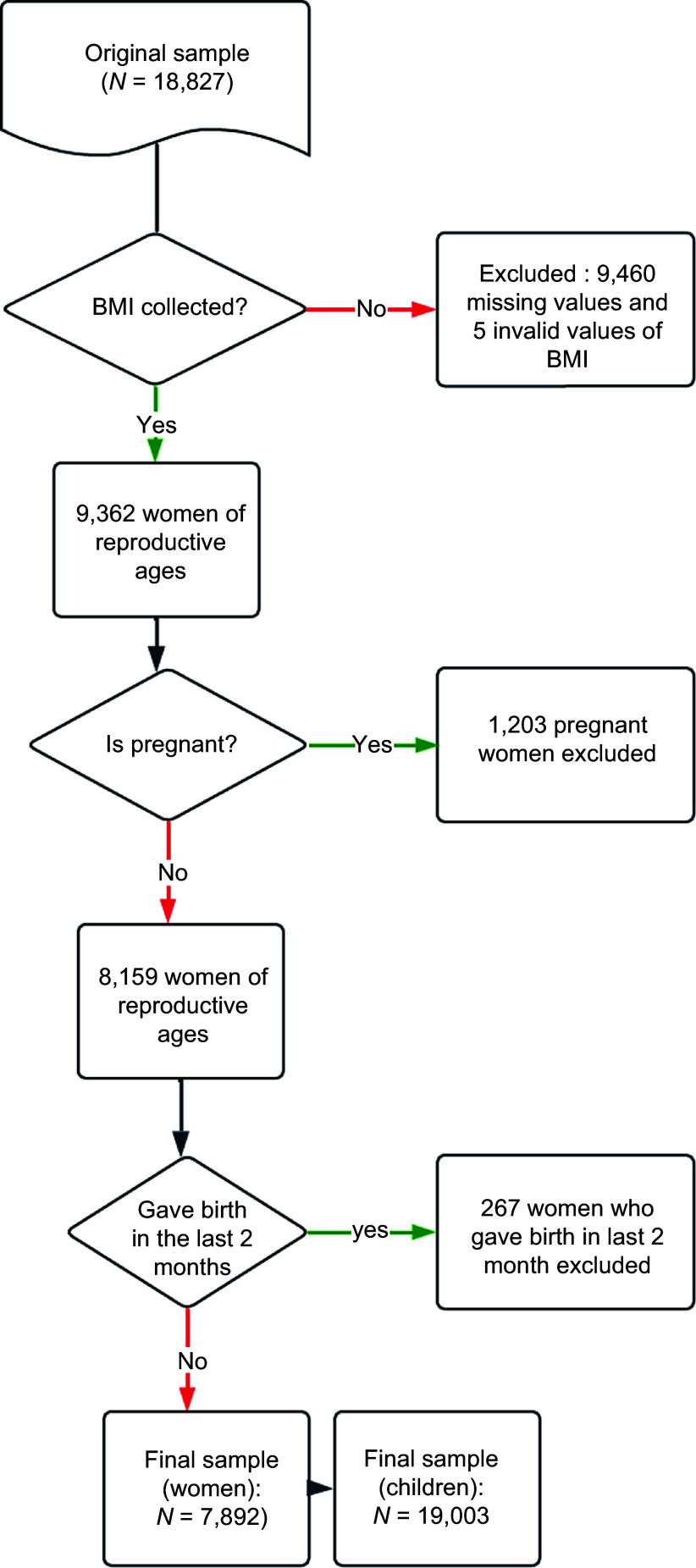
Selection of the final sample.

Analyses are restricted to women for whom BMI was collected (see Fig. 1). Likewise, analyses for child health outcomes are restricted to children from women with valid information on BMI.

### Ethics statement

Ethical approvals were obtained from the national ethics committees of DRC before the surveys were conducted. Written informed consent was obtained from every participant before she/he was allowed to take part in the survey. Consent was obtained from parents before their children’s measurements were taken. The DHS Program in the USA granted the authors permission to use the data. The data were completely anonymous; therefore, the authors did not seek further ethical clearance at the university since the data are publicly available at https://dhsprogram.com/data/available-datasets.cfm.

## Variables measurement

### Outcomes

#### Maternal nutritional status

To estimate the prevalence of overweight and obesity among women of reproductive ages, original information was obtained from the BMI derived from results of height and weight measurements. Trained field technicians collected the height and weight using standard techniques^(29)^. Using electronic Seca scales with a digital screen, women’s weight were measured, while height measurements were taken using a stadiometer produced by Shorr Productions. BMI, referred to as Quetelet’s Index^(30)^, was derived by dividing weight in kilograms by the squared height in metres. Based on the BMI ( 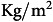 ) estimates, and according to WHO guidelines for SSA, the participants were classified as underweight (BMI < 18·50 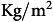 ), normal weight (18·50–24·99 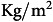 ), overweight (25·0–29·9 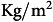 ) and obese (>= 30·0 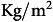 ). Preliminary analyses showed that obesity was marginal in the DRC. Therefore, and to avoid instability of statistical models, overweight and obesity were grouped in the category overweight/obesity, resulting into three categories. The normal weight (18·50–24·99 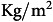 ) was used as reference category in the analysis.

### Child health outcomes

Previous literature has extensively documented the relationship between maternal nutritional status, referred to as **maternal overweight/obesity** and child health outcomes^(11,12,21–24)^. This paper is interested in the associations between maternal overweight/obesity and (*i*) neonatal mortality; (*ii*) post-neonatal mortality; (*iii*) infant mortality; (*iv*) childhood mortality and (*v*.) under-five mortality. All these indicators have been linked to development levels worldwide^(31)^ with developing countries experiencing higher levels of child mortality compared with developed countries^(32,33)^.

DHS collect information about age at death for deceased children. This information was used to classify the *period of child deaths* for deceased children. All these variables are binary, indicating whether a child has deceased in a specific age interval. These include:

- *Neonatal mortality* (nn): The risk of dying before birth and first month.
- *Post-neonatal mortality* (pnn): The risk of dying between first month and 11 months, contingent to surviving the first month.
- *Infant mortality* (IM): The risk of dying between birth and 11 months.
- *Childhood mortality* ( 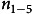 ): The risk of dying between first year and before 5 years, for children who survived till the first anniversary.
- *Under-five mortality* (U5M): The risk of dying between birth and the fifth birthday.

### Key independent variables

In this paper, two set of independent variables, selected from existing literature, were used to estimate the associations between maternal nutritional status and child health outcomes. Given the nature of analyses, the unit of analysis was different for each set of estimations. For maternal nutritional status, *woman* was the unit of analysis while *child* was the unit of analysis to estimate the associations between maternal nutritional status and child mortality.

#### Maternal overweight/obesity

To estimate maternal nutritional status, the predictors used in the analysis included women’s age and education, working status at the time of survey, breast-feeding status, marital status, sex of the head of household, household wealth index, place of residence and province of residence. The original variable household wealth index has five categories (poorest, poor, middle, rich and richest). In this study, this variable was recorded into two categories: poor (poorest and poor) and non-poor households.

#### Child health outcomes

The predictors of interest to estimate the risk of dying in a specific age interval included **maternal nutritional status** as key independent variable, controlling for sex of the child, pregnancy outcome (child is singleton), birth order, women’s age and education, parity, working status, sex of head of household, household wealth index, place of residence and province of residence.

### Analytical strategy

#### Maternal nutritional status

The paper used multinomial logistic regression (MLR) to estimate the probabilities of women’s nutritional status. MLR approach is appropriate since the outcome measure is polychotomous. Further, MLR was considered attractive analytical technique because it does not assume normality, linearity or homoscedasticity^(34)^. In MLR, vectors 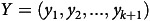 are observed for the dependent variable; 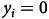 for all 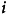 , and one 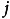 with 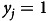 , and corresponding *probability* 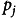 . The MLR is given by:
(1)

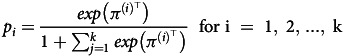

and
(2)

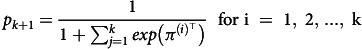

where 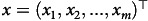 is the vector of covariates and 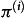 is the parameter vector corresponding to the *i-th* response category. In equation (2), the parameters set to zero and allows computing the probability for the base category in the MLR. The MLR model was performed to investigate the relationship between maternal age at the time of survey and nutritional status, controlling for other relevant variables. Using a BMI category of 18.5–24.99 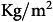 (*normal weight*) as the reference category, a set of logistic regressions for underweight and overweight/obese were estimated. All covariates were simultaneously entered into the model. Results were presented in the form of coefficients with significance levels and 95 % CI.

*Child mortality*. In this paper, the indicators pertaining to child health outcomes were all defined as *binary outcomes*. In a previous study, scholars used *linear probability model (LPM)* to estimate the effects of dying before the first anniversary^(35)^. Although this statistical technique leads to easy interpretation, it does not account for the non-linearity of the events; yet, demographic research has clearly pointed out to this issue^(36)^. Therefore, *generalised linear models (GLM)* were used in this paper to account for non-linearity of the outcomes. In GLM, outcomes ( 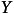 ) are assumed to be generated from a distribution belonging to a large family of *exponential distributions*. Under this assumption, the conditional mean 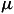 of the distribution of the outcomes depends on the independent variables 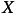 as follows:
(3)

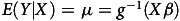




Compared with the *standard linear model*, GLM have the following advantages: (*i*) dropping the normality requirements; (*ii*) relaxing the homoscedasticity assumption and (*iii*) allowing for some function of 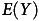 to be linear in the parameters as a **link** 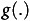 .

Specifically, estimations in this paper were performed using the family *binomial* and *logit* as the **link function** in STATA 18 SE. Additionally, the number of iterations was increased using the *option* 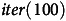 to improve the stability of the models.

## Results

### Descriptive results

Tables 1–3 present descriptive results for samples of women of reproductive ages (Tables 1 and 2) and children under age 5 years (Table 3), respectively. Findings indicate that, overall, 16 % of women of reproductive ages are overweight or obese (Table 1). A marginal percentage (result not shown) of them are obese (3·4 %). There are rationales to pay attention to geographical variations of maternal nutritional status given diet differences across provinces in the country. In this paper, Table 2 highlights the importance of geographical variations in analysing women’s weight. Clearly, women’s average weight varies by province, urban residence and household poverty levels. Indeed, the average weight of women of reproductive ages ranged from 48·5 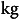 in Kongo Central to 61·3 kg in Kinshasa. Findings also showed rural–urban differences (51·1 kg *v*. 58·4 kg), differences by poverty levels, with women living in better-off households being heavier (on average: 56·2 kg) compared with their counterparts living in poor households (49·9 kg).

**Table 1.** Descriptive statistics of the sample of women of reproductive ages

Dependent variable: Women’s nutritional status	*n* (unweighted)	Weighted %/Mean	sd
Underweight	1197	14·4	
Normal weight	5587	69·7	
Overweight/obese	1108	16·0	
**Independent variables**			
**Women’s level variables**			
Age	7892	28·5	9·9
Education (in completed years)	7892	6·1	4·1
Working status (% working at survey)	5318	66·5	
Breast-feeding (% women breast-feeding at survey)	3117	38·3	
Marital status			
Single	4545	29·3	
Married/cohabiting	12 448	59·4	
Widowed/divorced/separated	1834	11·3	
**Household- and community-level variables**			
Household head is male	5798	73·9	
Household wealth index			
Poor (40 % bottom)	3353	37·2	
Non-poor (60 % top)	4539	62·8	
Urban residence (% urban)	2882	37·7	
Province of residence			
Kinshasa	769	12·0	
Kongo Central	1043	16·1	
Bandundu	414	4·9	
Equator	1098	12·8	
Kasai Occidental	594	6·4	
Kasai Oriental	893	10·2	
Katanga	896	8·9	
Maniema	362	3·1	
North Kivu	510	9·4	
Orientale	895	9·5	
South Kivu	418	6·7	
**Total sample size**	**7892**	**100·0**	

**Table 2.** Sub-national estimates of maternal nutritional status among women of reproductive ages: making a case for a geographical inquiry

Variables	Weight (in kg)		Height (in cm)		BMI (in kg/m^2^)	
	Mean	95 % CI	Mean	95 % CI	Mean	95 % CI
**Province of residence**						
Kinshasa	61·3	(60·2, 62·4)	160·7	(159·9, 161·5)	23·7	(23·4, 24·1)
Kongo Central	48·5	(47·5, 49·5)	155·1	(153·9, 156·3)	20·1	(19·8, 20·4)
Bandundu	51·0	(47·9, 54·1)	155·8	(154·6, 157·0)	20·9	(19·9, 21·9)
Equator	53·7	(52·4, 54·9)	158·5	(157·7, 159·3)	21·3	(21·0, 21·6)
Kasai Occidental	52·4	(51·0, 53·7)	157·8	(156·9, 158·8)	21·0	(20·6, 21·3)
Kasai Oriental	53·8	(52·1, 55·6)	158·5	(157·2, 159·9)	21·3	(20·9, 21·7)
Katanga	52·5	(50·5, 54·4)	155·4	(154·4, 156·4)	21·6	(21·1, 22·1)
Maniema	53·1	(51·6, 54·7)	154·1	(151·8, 156·4)	22·4	(21·8, 22·9)
North Kivu	55·7	(54·0, 57·5)	153·2	(151·9, 154·4)	23·7	(23·2, 24·2)
Orientale	55·2	(53·6, 56·7)	155·7	(154·1, 157·4)	22·7	(22·3, 23·1)
South Kivu	54·9	(51·5, 58·4)	153·6	(151·7, 155·5)	23·2	(22·3, 24·0)
**Residence**						
Rural	51·1	(50·3, 51·8)	155·2	(154·7, 155·7)	21·2	(20·9, 21·4)
Urban	58·4	(57·7, 59·2)	158·8	(158·3, 159·2)	23·1	(22·9, 23·4)
**Household wealth index**						
Poor	49·9	(49·4, 50·4)	155·0	(154·4, 155·6)	20·8	(20·6, 20·9)
Non-Poor	56·2	(55·5, 56·9)	157·4	(157·0, 157·9)	22·6	(22·3, 22·8)

**Table 3.** Maternal nutritional status and child mortality in the Democratic Republic of the Congo

Women’s nutritional status	Unweighted N	Neonatal mortality	95 % CI
Underweight	2612	35·2	(25·0, 49·4)
Normal weight	13 581	30·3	(26·1, 35·2)
Overweight/obese	2810	23·4	(17·4, 31·4)
Total	19 003	29·7	(26·1, 33·7)
**Women’s nutritional status**	**Unweighted N**	**Post-neonatal mortality**	**95 % CI**
Underweight	2534	48·6	(35·1, 66·9)
Normal weight	13 184	42·8	(37·4, 49·0)
Overweight/obese	2744	40·0	(29·0, 54·9)
Total	18 462	43·0	(38·0, 48·7)
**Women’s nutritional status**	**Unweighted N**	**Infant mortality**	**95 % CI**
Underweight	2612	82·0	(64·4, 104·0)
Normal weight	13 581	71·9	(64·5, 80·0)
Overweight/obese	2810	62·5	(48·8, 79·7)
Total	19 003	71·4	(64·4, 79·1)
**Women’s nutritional status**	**Unweighted N**	**Child mortality**	**95 % CI**
Underweight	2410	50·7	(38·8, 66·0)
Normal weight	12 615	44·3	(37·9, 51·8)
Overweight/obese	2643	49·2	(38·0, 63·5)
Total	17 668	46·0	(41·1, 51·5)
**Women’s nutritional status**	**Unweighted N**	**Under-five mortality**	**95 % CI**
Underweight	2612	128·6	(107·1, 153·6)
Normal weight	13 581	113·0	(104·2, 122·5)
Overweight/obese	2810	108·6	(88·2, 133·1)
Total	19 003	114·2	(105·4, 123·5)

Regarding height, findings indicate that shortest women live in the provinces of North Kivu (average: 153·2 cm), South Kivu (average: 153·6 cm) and Maniema (average: 154·1 cm). In contrast, tallest women are found in Kinshasa, the Capital City, with an average of 160·7 cm. Similarly, findings indicate differences between rural and urban areas, as well as between poor and non-poor households. Finally, analyses of BMI showed that skinniest women lived in the province of Kongo Central (on average: 20·1 Kg/ 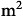 ). Again, women living in the provinces of North Kivu, South Kivu and Maniema recorded highest BMI, along with Kinshasa.

Table 3 focuses on the associations between women’s nutritional status and child mortality. Figures in Table 3 are expressed in terms of **number of deaths per 1000 live births**. Findings indicate a clear gradient between maternal nutritional status and children health outcomes. Indeed, child health outcomes were worst among children born from underweight women compared with those born from overweight/obese women. However, these differences were not statistically significant. Neonatal mortality was higher among children born from underweight women (35·2 p. 1000) compared with those born from overweight/obese women (23·4 p.1000). Post-neonatal mortality rates were also higher among children born from underweight women (48·6 p.1000) compared with those from overweight/obese women (40·0 p.1000). Findings also that infant mortality rates were higher among children born from underweight women (82·0 p.1000) compared with those from overweight/obese women (62·5 p.1000). Likewise, childhood mortality rates were higher among children born from underweight women (50·7 p.1000) compared with those from overweight/obese women (49·2 p.1000). Finally, under-five mortality rates were higher among children born from underweight women (128·6 p.1000) compared with those from overweight/obese women (108·6 p.1000).

### Multivariate results

#### Predicting maternal nutritional status

**Figure 2.**
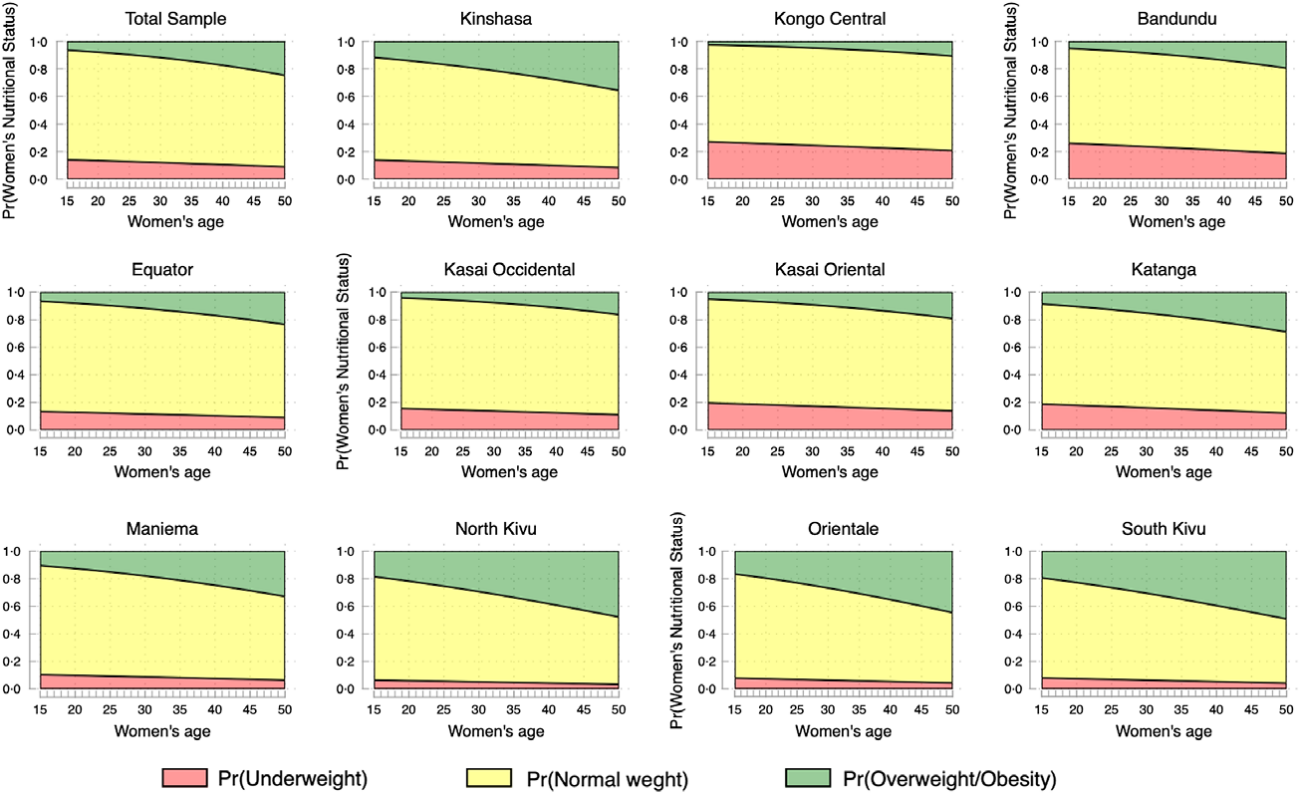
Predicted probabilities of women’s nutritional status at national and sub-national levels in the Democratic Republic of Congo.

**Figure 3.**
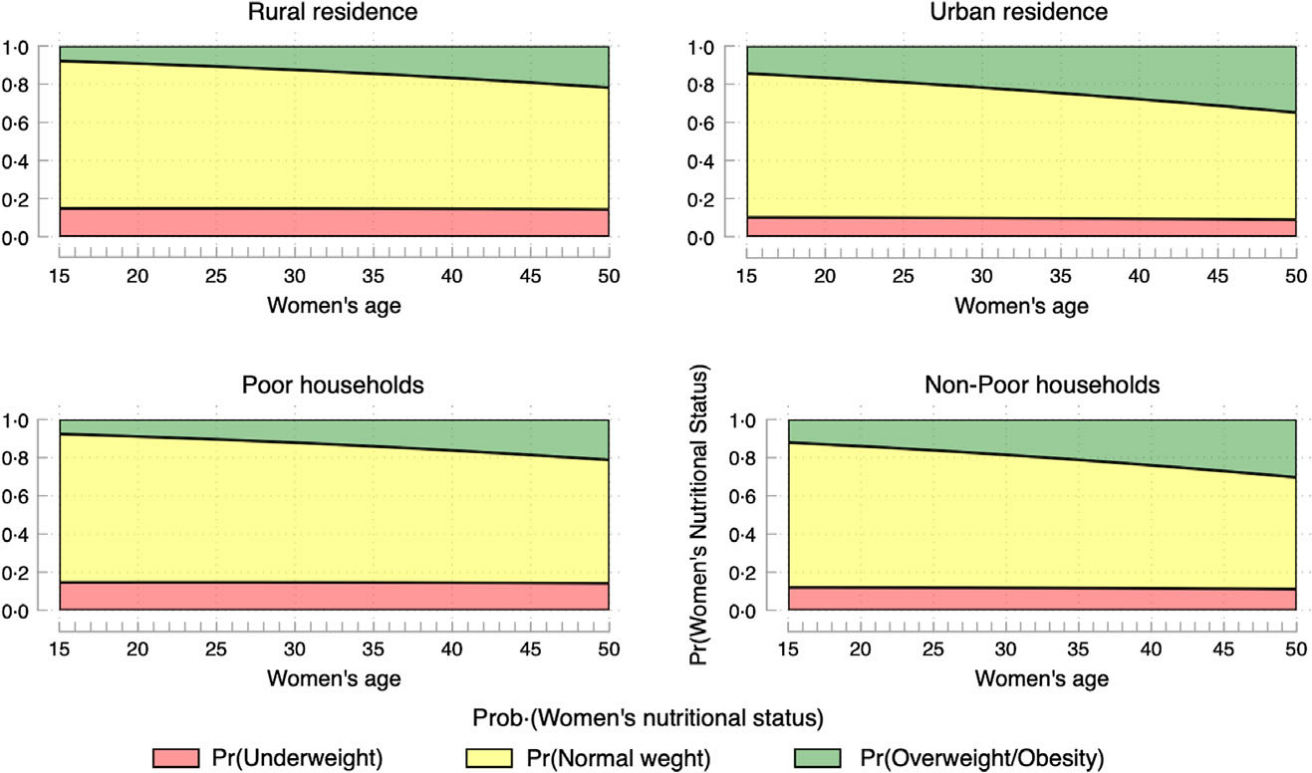
Predicted probabilities of women’s nutritional status in the Democratic Republic of Congo, by place of residence and household poverty levels.

Figures 2 and 3 display the predicted probabilities of the nutritional status of women of reproductive ages in the Democratic Republic of Congo, based on a MLR of women’s observed nutritional status on age, while controlling for women’s education, working status, breast-feeding status, marital status, parity, sex of household head, household poverty, place of residence and provinces.

When focusing on overweight/obesity only (Fig. 2), the following provinces are the most affected: North Kivu and South Kivu, Orientale, and to some extent, Kinshasa. Marginally, the provinces of Kasai oriental, Bandundu and Katanga are affected. Figure 3 shows that obesity is primarily concentrated in urban areas and among better-off households.

#### Associations between maternal nutritional status and child mortality

**Figure 4.**
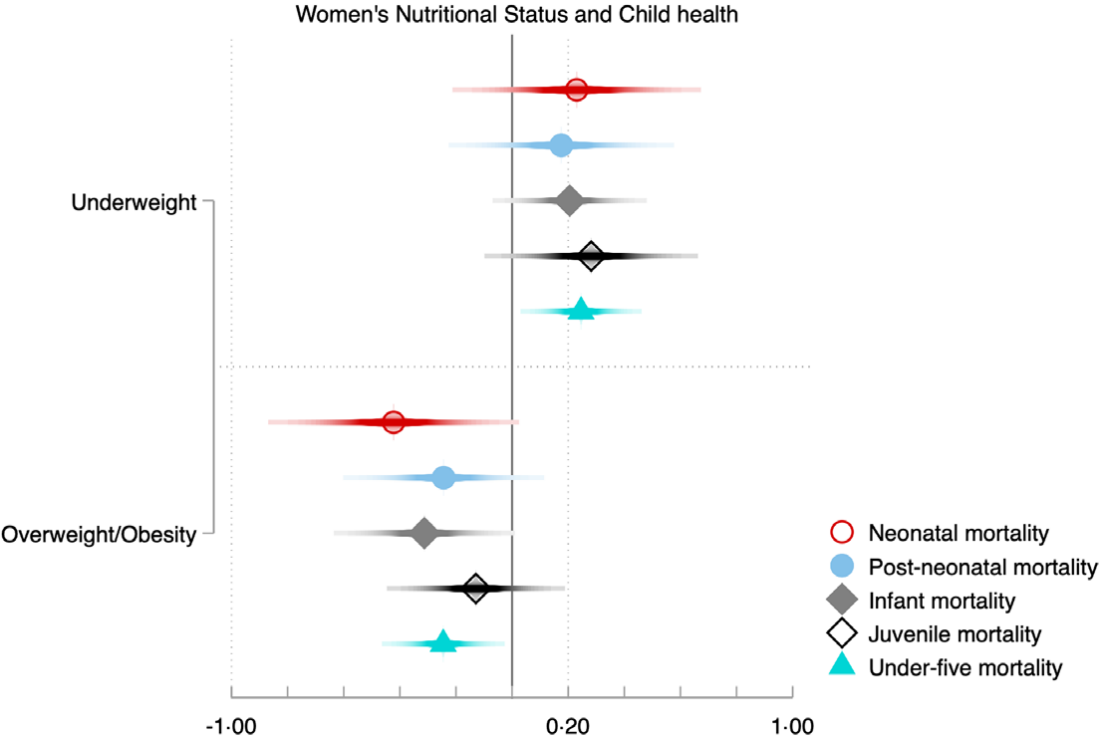
Associations between women’s nutritional status and child mortality in the Democratic Republic of Congo.

Figure 4 and Table 4 present the associations between women’s nutritional status (for the entire sample, and by household poverty levels and place of residence) and five child health outcomes, including neonatal mortality, post-neonatal mortality, infant mortality, child mortality, and under-five mortality, controlling for child-level variables (sex of the child, birth order and type of birth), woman-, household-, and community-level variables (age, education, parity, sex of household head, household poverty, place of residence and province).

**Table 4.** Maternal nutritional status and child mortality: entire sample, and by household poverty levels and place of residence

Panel A. Entire sample (children under 5 years)					
	Neonatal mortality	Post-neonatal mortality	Infant mortality	Childhood mortality	Under-five mortality
Women’s nutritional status (ref.: normal weight)					
Underweight	0·228	0·174	0·203*	0·301**	0·252***
	(–0·113, 0·569)	(–0·133, 0·480)	(–0·006, 0·411)	(0·012, 0·591)	(0·087, 0·417)
Overweight/obesity	–0·358**	–0·219	–0·279**	0·040	–0·155**
	(–0·631, –0·084)	(–0·488, 0·049)	(–0·498, –0·059)	(–0·199, 0·279)	(–0·303, –0·007)
**Panel B. Household poverty level**					
**Poor households**	Neonatal mortality	Post-neonatal mortality	Infant mortality	Childhood mortality	Under-five mortality
Women’s nutritional status (ref.: normal weight)					
Underweight	0·0899	0·187	0·157	0·138	0·159
	(–0·344, 0·524)	(–0·149, 0·522)	(–0·119, 0·434)	(–0·236, 0·513)	(–0·078, 0·397)
Overweight/Obesity	–0·395	–0·131	–0·24	–0·169	–0·223
	(–1·010, 0·221)	(–0·662, 0·400)	(–0·700, 0·220)	(–0·502, 0·164)	(–0·535, 0·088)
**Non-poor households**	Neonatal mortality	Post-neonatal mortality	Infant mortality	Childhood mortality	Under-five mortality
Women’s nutritional status (ref.: normal weight)					
Underweight	0·36	0·177	0·258*	0·485**	0·357***
	(–0·134, 0·853)	(–0·272, 0·626)	(–0·022, 0·538)	(0·105, 0·864)	(0·135, 0·579)
Overweight/obesity	–0·273*	–0·200	–0·232*	0·141	–0·087
	(–0·574, 0·027)	(–0·512, 0·112)	(–0·473, 0·008)	(–0·185, 0·467)	(–0·259, 0·086)
**Panel C. Place of residence**					
**Rural women**					
	Neonatal mortality	Post-neonatal mortality	Infant mortality	Childhood mortality	Under-five mortality
Women’s nutritional status (ref.: normal weight)					
Underweight	0·190	0·181	0·193*	0·299*	0·247***
	(–0·185, 0·565)	(–0·162, 0·524)	(–0·034, 0·419)	(–0·025, 0·623)	(0·061, 0·432)
Overweight/obesity	–0·503**	–0·285	–0·380**	0·0613	–0·210**
	(–0·886, –0·120)	(–0·655, 0·0852)	(–0·699, –0·0611)	(–0·279, 0·402)	(–0·398, –0·022)
**Urban women**					
	Neonatal mortality	Post-neonatal mortality	Infant mortality	Childhood mortality	Under-five mortality
Women’s nutritional status (ref.: normal weight)					
Underweight	0·191	0·150	0·169	0·227	0·189
	(–0·403, 0·786)	(–0·313, 0·613)	(–0·171, 0·509)	(–0·283, 0·736)	(–0·068, 0·446)
Overweight/obesity	–0·207	–0·158	–0·18	0·0619	–0·0876
	(–0·642, 0·229)	(–0·527, 0·210)	(–0·480, 0·119)	(–0·266, 0·390)	(–0·330, 0·155)

CI are in parentheses.

Statistical significance: ***P < 0.01, **P < 0.05, *P < 0.1.

Figure 4 confirms the absence of a *clear gradient* between maternal nutritional status and child health outcomes as aforementioned. For instance (see Table 4), underweight is *positively* associated with infant mortality ( 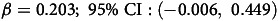 ), childhood mortality ( 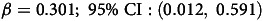 ) and U5M ( 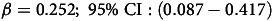 ). Overweight/obesity is *negatively* associated with three child health outcomes, including neonatal mortality ( 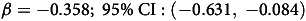 ), infant mortality ( 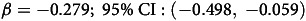 ) and under-five mortality ( 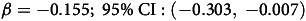 ).

When data are disaggregated by household poverty levels (see Table 4; Panel B), findings show that women’s nutritional status is not significantly associated with child health outcomes for poor households. In advantaged households, some associations between women’s nutritional status and child health outcomes reached statistical significance, mimicking the pattern in the entire sample. Indeed, underweight was *positively* associated with infant mortality ( 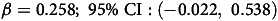 ), childhood mortality ( 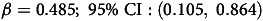 ) and under-five mortality ( 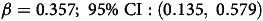 ). Likewise, overweight/obesity was marginally *negatively* associated with neonatal mortality ( 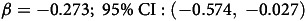 ) and infant mortality ( 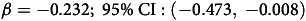 ).

Looking into the associations between maternal nutritional status and child health outcomes by place of residence (Table 4; Panel C). Among rural women, underweight was *positively* associated, net of controls, with under-five mortality ( 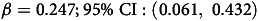 ) and marginally with infant mortality ( 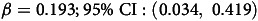 ) and childhood mortality ( 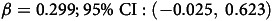 ). In contrast, overweight/obesity was *negatively* associated with neonatal mortality ( 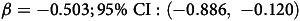 ), infant mortality ( 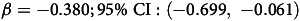 ) and under-five mortality ( 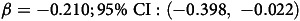 ). Among urban women, no significant associations are found between maternal nutritional status and child health outcomes.

#### Geographical variations of the associations between maternal nutritional status and child health outcomes

Table 5 presents the associations between maternal nutritional status and child health outcomes by province of residence. Although Table 5 provides much more details, this section focuses on overweight/obesity. In Bandundu, findings indicated that maternal overweight/obesity was *significantly and negatively* associated with childhood mortality ( 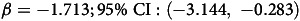 ) and under-five mortality ( 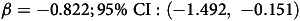 ). In Equator, being overweight/obese was *significantly and positively* associated with under-five mortality ( 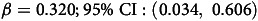 ). In contrast, being overweight/obese in North Kivu was *significantly and negatively* associated with under-five mortality (*β* = −0.608; 95% *CI* : (−1.202, −0.014))

**Table 5.** Maternal nutritional status and child mortality, sub-national estimates

Women’s nutritional status (ref.: normal weight)					
Kinshasa	Neonatal mortality	Post-neonatal mortality	Infant mortality	Childhood mortality	Under-five mortality
Underweight	(n.a)	1·304***	0·622*	(n.a)	0·0765
	…	(0·632, 1·977)	(–0·0539, 1·298)	…	(–0·548, 0·701)
Overweight/obesity	–0·658	–0·235	–0·397	–0·0561	–0·261
	(–1·467, 0·150)	(–0·894, 0·424)	(–0·998, 0·205)	(–0·648, 0·535)	(–0·736, 0·214)
**Kongo Central**	**Neonatal mortality**	**Post-neonatal mortality**	**Infant mortality**	**Childhood mortality**	**Under-five mortality**
Underweight	0·224	–0·546*	–0·187	0·563**	0·112
	(–0·608, 1·056)	(–1·162, 0·069)	(–0·637, 0·263)	(0·012, 1·115)	(–0·257, 0·481)
Overweight/obesity	–0·748	–0·241	–0·358	–1·247*	–0·623*
**Bandundu**	**Neonatal mortality**	**Post-neonatal mortality**	**Infant mortality**	**Childhood mortality**	**Under-five mortality**
Underweight	–0·205	0·497	0·280	0·456	0·378
	(–1·539, 1·128)	(–0·632, 1·627)	(–0·557, 1·116)	(–0·883, 1·794)	(–0·298, 1·054)
Overweight/obesity	–0·149	–0·761*	–0·500*	–1·713**	–0·822**
	(–0·936, 0·638)	(–1·633, 0·111)	(–1·087, 0·0858)	(–3·144, –0·283)	(–1·492, –0·151)
**Equator**	**Neonatal mortality**	**Post-neonatal mortality**	**Infant mortality**	**Childhood mortality**	**Under-five mortality**
Underweight	0·491*	0·216	0·333*	0·713***	0·537***
	(–0·052, 1·034)	(–0·338, 0·771)	(–0·036, 0·702)	(0·206, 1·220)	(0·207, 0·867)
Overweight/obesity	0·456	0·348	0·406*	0·125	0·320**
	(–0·107, 1·020)	(–0·345, 1·041)	(–0·057, 0·869)	(–0·547, 0·797)	(0·034, 0·606)
**Kasai Occidental**	**Neonatal mortality**	**Post-neonatal mortality**	**Infant mortality**	**Childhood mortality**	**Under-five mortality**
Underweight	0·571	0·411	0·490	–0·494*	0·0632
	(–0·340, 1·482)	(–0·518, 1·341)	(–0·157, 1·138)	(–1·009, 0·022)	(–0·387, 0·513)
Overweight/obesity	0·048	–0·279	–0·144	–0·012	–0·041
	(–1·212, 1·307)	(–1·336, 0·778)	(–1·060, 0·772)	(–0·348, 0·325)	(–0·479, 0·396)
**Kasai Oriental**	**Neonatal mortality**	**Post-neonatal mortality**	**Infant mortality**	**Childhood mortality**	**Under-five mortality**
Underweight	0·257	0·513	0·423	0·277	0·371*
	(–0·609, 1·124)	(–0·687, 1·714)	(–0·286, 1·132)	(–0·697, 1·251)	(–0·032, 0·774)
Overweight/obesity	–0·55	0·139	–0·0635	–0·161	–0·104
	(–1·549, 0·449)	(–0·712, 0·990)	(–0·898, 0·771)	(–0·867, 0·544)	(–0·726, 0·519)
**Katanga**	**Neonatal mortality**	**Post-neonatal mortality**	**Infant mortality**	**Childhood mortality**	**Under-five mortality**
Underweight	–0·373	0·247	–0·0232	–0·018	–0·0343
	(–1·125, 0·379)	(–0·404, 0·898)	(–0·540, 0·494)	(–0·528, 0·492)	(–0·439, 0·371)
Overweight/obesity	–0·916***	0·194	–0·274	0·679*	0·0863
	(–1·529, –0·304)	(–0·491, 0·878)	(–0·789, 0·242)	(–0·112, 1·471)	(–0·345, 0·517)
**Maniema**	**Neonatal mortality**	**Post-neonatal mortality**	**Infant mortality**	**Childhood mortality**	**Under-five mortality**
Underweight	0·839	0·981*	0·903**	0·091	0·744
	(–0·421, 2·099)	(–0·167, 2·129)	(0·021, 1·786)	(–1·366, 1·547)	(–0·284, 1·771)
Overweight/obesity	–1·079	–0·299	–0·71	0·676	–0·0706
	(–3·006, 0·848)	(–1·508, 0·910)	(–2·238, 0·818)	(–0·274, 1·625)	(–0·710, 0·568)
**North Kivu**	**Neonatal mortality**	**Post-neonatal mortality**	**Infant mortality**	**Childhood mortality**	**Under-five mortality**
Underweight	(n.a)	–0·768	–1·590*	1·362**	0·319
	…	(–2·730, 1·193)	(–3·339, 0·158)	(0·252, 2·472)	(–0·737, 1·376)
Overweight/obesity	–0·300	–0·854	–0·558*	–0·685	–0·608**
	(–0·994, 0·393)	(–1·911, 0·203)	(–1·193, 0·076)	(–1·589, 0·220)	(–1·202, –0·014)
**Orientale**	**Neonatal mortality**	**Post-neonatal mortality**	**Infant mortality**	**Childhood mortality**	**Under-five mortality**
Underweight	0·091	0·174	0·136	–0·116	0·039
	(–0·839, 1·020)	(–0·623, 0·970)	(–0·373, 0·644)	(–0·835, 0·604)	(–0·438, 0·518)
Overweight/obesity	–0·803*	–0·313	–0·537*	0·361	–0·124
	(–1·683, 0·078)	(–1·150, 0·524)	(–1·156, 0·082)	(–0·135, 0·858)	(–0·634, 0·386)
**South Kivu**	**Neonatal mortality**	**Post-neonatal mortality**	**Infant mortality**	**Childhood mortality**	**Under-five mortality**
Underweight	0·828**	0·600	0·764***	–0·726	0·556***
	(0·193, 1·462)	(–0·485, 1·686)	(0·437, 1·091)	(–2·288, 0·836)	(0·245, 0·867)
Overweight/obesity	0·0804	–0·0521	0·005	0·303	0·125
	(–0·519, 0·680)	(–1·091, 0·986)	(–0·718, 0·728)	(–0·992, 1·599)	(–0·281, 0·530)

n.a.: Estimates not available due to small cell sizes.

CI are in parentheses.

Statistical significance: ***P < 0.01, **P < 0.05, *P < 0.1.

## Discussion

The objectives of the paper are twofold. First, the study investigated the relationship between maternal age and maternal nutritional status, with a special attention on obesity in the Democratic Republic of Congo. Second, the paper was interested in the relationship between maternal nutritional status and child mortality assuming that children from obese mothers have worse outcomes compared with those born from non-obese mothers.

### Main findings

#### Maternal age and nutritional status among women of reproductive ages

Findings from this paper showed that the likelihood of being obese among women of reproductive ages in the DRC increased with age, after controlling for women’s education, working status, breast-feeding status, marital status, parity, sex of household head, household poverty, place of residence and province. However, the percentage of obese women was marginal (3·4 %) and lower than reported in previous studies. For instance, a study reported that 13 % of adults aged 18 years and above were obese, and 39 % were overweight^(37)^. Collapsing overweight and obesity brings up to 16 %; this percentage became comparable to figures reported in Uganda around 2011^(38)^ and SSA^(39)^. The study in Uganda reported a prevalence of overweight/obesity of 19·4 % in 2011. Furthermore, obesity was more prevalent among women living in advantaged households^(40)^. Explanations include the lifestyles and physical activity. Women from advantaged households have more access to processed foods; therefore, increasing their risks to be obese. Also, they do adopt a much more sedentary lifestyle. They do exercise less compared with women from poor households and rural areas. For instance, they have access to maids and personal drivers which significantly limit their opportunities to exercise. In contrast, women from disadvantaged households and rural areas have more opportunities to exercise due to financial constraints and lifestyle, therefore, decreasing the likelihood of being obese.

The paper highlighted geographical variations of women’s nutritional status in the Democratic Republic of Congo. Overall, all the eleven provinces are unevenly affected in terms of the prevalence of obesity. Some provinces (North Kivu, Orientale and South Kivu) and to some extent Kinshasa the Capital City are more affected. These inequalities will significantly impact the Sustainable Development Goals (SDG) related to obesity. SDG 2 and 3 focus on ending all forms of malnutrition (SDG 2) and NCD (SDG 3), particularly as the country grapples with inequalities in obesity prevalence. Therefore, the government should devise more effective programmes and interventions to tackle these inequalities to ensure that the country could reach SDG goals. Indeed, DRC has made limited progress towards achieving the diet-related NCD targets^(18)^.

Figures reported here on overweight/obesity are lower than those found in more localised studies^(41)^. In a baseline study on obesity, diabetes and hypertension among Tenke Fungurume Mining workforce, findings showed that prevalence of obesity increased from 4·5 % to 11·1 % between 2010 and 2015. Localized studies could uncover further disparities across provinces, highlighting the need for urgent action to address this pandemic and reduce the prevalence of NCD in the country.

#### Maternal nutritional status and child mortality

Previous studies have consistently reported that maternal obesity is positively and significantly with adverse pregnancy outcomes^(11,24)^ and health risks during infancy^(26,42–45)^. This study provided partial support of the associations between maternal obesity and child mortality. In the entire sample, maternal obesity was positively and significantly associated with childhood mortality. When data are disaggregated by province, urban residence and poverty levels, findings are more inconsistent compared with what has been reported in previous studies.

Two empirical explanations can be drawn from previous studies. First, previous have been undertaken in developed countries where obesity prevalence is alarmingly high and could lead to adverse pregnancy and child outcomes. Indeed, the average national prevalence of obesity in developed countries is estimated at 17 %^(5)^. The corresponding figure in developing countries ranges between 6·8 % and 8·7 %^(46,47)^. In the current study, the prevalence of obesity among women of reproductive ages was very low. That can explain the poor and unstable relationship observed between maternal obesity and child obesity for the entire sample and disaggregated data. Second, the concentration of obesity among women of reproductive ages can be another plausible explanation. In developing countries and SSA, previous studies showed that the prevalence of obesity is low and concentrated among advantaged women^(40)^.

Additionally, previous studies showed that child mortality is higher in poor households. Therefore, it might be a *compensation effect* concerning the associations between maternal obesity and child mortality. While one might expect maternal obesity to be associated with child mortality; being in better-off households provides with a buffer in the sense that those households provide children with good care when they are sick and therefore leads to lower risks of child mortality. This explanation is not definitive because the relationship between maternal obesity and child mortality is not widely documented in SSA. Some exceptions do exist^(48–50)^. However, these studies mostly used pooled data, therefore, increasing statistical power of the estimates and masking differences within and across countries. The current study dug into geographical disparities in maternal obesity and how this might affect child mortality. With this approach, the paper provided insights to better devise national and local strategies to better tackle the double burden of nutrition in the country.

### Study strengths and limitations

The paper used nationally representative data which yielded to robust estimates of the associations between maternal age and obesity on the one hand, and on the other hand, of the associations between maternal nutritional status and child mortality. Additionally, women’s height and weight data used to compute BMI were objectively measured, reducing possible misclassification. However, all women in the original sample were not included in the analyses because BMI was not collected. This reduced statistical power of modelling, especially at sub-national levels.

### Conclusion and policy implications

The paper evidenced the disparities of overweight/obesity prevalence in the Democratic Republic of Congo. The consequences of overweight/obesity on population health are poorly documented developing countries, while it is alarmingly increasing in SSA. Therefore, more comprehensive and effective national and sub-national interventions to tackle overweight and obesity in the country are of chief importance to reach SDG 2 and 3. NCD have the potential to increase operational costs while decreasing productivity in the country and might bring additional pressure to health facilities.
